# 仪器分析课程中思政教学的探索与实践——以色谱课程教学为例

**DOI:** 10.3724/SP.J.1123.2024.04005

**Published:** 2024-09-08

**Authors:** Yida ZHANG, Haixia ZHANG

**Affiliations:** 兰州大学化学化工学院,甘肃兰州 730000; College of Chemistry and Chemical Engineering, Lanzhou University, Lanzhou 730000, China

**Keywords:** 课程思政, 仪器分析, 色谱, 实践, 设计, course ideology and politics, instrumental analysis, chromatography, practice, design

## Abstract

仪器分析是化学专业的重要专业基础课程之一,其教学内容丰富多样且与日常生活等密切相关,蕴含了大量的思政资源。同时该课程也是培养德智体美全面发展的社会主义建设者和接班人的重要途径。本文以仪器分析中的色谱技术为例,从色谱知识回顾、溶液配制、标准操作和指导实验等方面入手,深入挖掘思政育人元素,将其有机融入仪器分析课程教学中。在教授专业知识的同时,增加了知识的多元性和教学内容的丰富性,启发并增强了学生的创新意识和理念,使学生树立人生目标和伟大理想,最终达到立德树人的目的。

## Abstract (en)

Instrumental analysis is an important professional course for many chemistry majors. Its teaching content is rich, diverse and is closely related to daily life. Moreover, this course contains a large number of ideological and political resources. This study investigates chromatography technology in instrumental analysis as an example, focusing on aspects such as chromatography knowledge, solution preparation, standard operations, and guided experiments, to deeply explore the elements of ideological and political education and organically integrate them into the teaching of instrumental analysis courses. Academic teaching of professional knowledge increases the diversity and richness of the teaching content and enhances the innovative skills of the students. In addition, this teaching method can inspire students to establish life goals and great ideals, with the aim of ultimately cultivating moral people.

新时代党和国家对教师提出了更高的要求,习总书记在全国高校思想政治工作会议上提出“教育强则国家强”。高等教育发展水平是一个国家发展水平和发展潜力的重要标志。实现中华民族伟大复兴,教育的地位和作用不可忽视。我们对高等教育的需要比以往任何时候都更加迫切,对科学知识和卓越人才的渴求比以往任何时候都更加强烈。党中央做出加快建设世界一流大学和一流学科的战略决策,目的是提高我国高等教育发展水平,增强国家核心竞争力^[1]^。因此,注重课程思政教育,坚持社会主义的办学方向,是践行上述要求与培养德智体美全面发展的社会主义建设者和接班人的重要途径。作为高校教师,不仅要通过榜样示范作用,增强思政课的感染力,还应注重挖掘榜样素材,让先进人物和事迹现身说法,发挥其示范作用。激励学生学习先进、靠近榜样,使抽象深奥的道理变得具体生动,转化为鲜活的素材,增强思政课的感染力。

兰州大学化学化工学院毕业生约75%继续深造,所以课程思政教育的开展不仅需要对学科专业知识教学中的思政资源进行深入发掘,还要充分发挥课堂优势实现育人目标。仪器分析是化学、药学和材料等学科学生的重要专业基础课程之一^[2]^,是使用复杂或特殊的仪器设备,通过测量物质的物理化学性质的参数变化获取物质化学组成、成分含量及化学结构等的一门学科。其课程特色是在基础化学知识上引进仪器设备,从常量、手动分析延伸至微量、自动化分析,教学内容丰富多样且与日常生活、社会发展和疾病健康等密切相关,蕴含了大量的思政资源^[3⇓-5]^。在仪器分析中,色谱是分离科学最常用的工具之一,也是仪器分析课程的重要章节,课程内容包括色谱知识基础、气相色谱和液相色谱;实验教学包括气相色谱、液相色谱、离子色谱、气相色谱-质谱联用;教学目标包括:(1)掌握色谱理论与流出曲线的参数计算;(2)掌握气相色谱和液相色谱的设备构造、方法建立及定量计算;(3)了解不同色谱方法的差异以及色谱未来发展的趋势;(4)提升科研思维能力和创新能力;(5)具有个体和学科服务社会的意识(见表1)。该课程不仅对学生思维模式的转变发挥重要作用,还引领学生树立理论知识和服务社会相结合的思政观念。兰州大学仪器分析课程是甘肃省一流课程,教师团队也是甘肃省高等学校省级教学团队。在备课、讲课、作业、考试等环节均进行了大量的研讨、沟通与设计,力争满足不同层次的学生需要^[6,7]^。如何在教学过程中挖掘并巧妙融入思政元素,将“知识传授”和“价值意识”融于一体,是任课教师面临的重要任务。

**表1:** 仪器分析课程思政与教学体系

序号	知识点	思政元素与教学设计	教学方法	对应正文第二段 的教学目标
1	色谱历史:次维特实验;马丁与辛格获得诺贝尔化学奖;中国西部色谱发展历程	从0到1以及科研加速度的辩证关系:科研是站在前人的肩膀上;科研要服务国家重大需求	类比:马拉松比赛	(4)(5)
2	流出曲线:分配系数与分配比;各种保留值;峰高和峰面积;三大参数计算与相互关系;色谱的两大理论	几率规律:理论向实验的转化策略;结果呈现方式;减小误差的策略;理论的呈现策略	启发:统计方法;比较法	(1)(4)
3	色谱仪器框架:各类检测器的特点;各类辅助器件的作用	科研思维模式:任何一个工具都不可能解决所有问题;没有任何一个方法是不能取代的;融于并服务社会才能发挥个人价值	功能导向推理	(2)(4)(5)
4	色谱方法的建立:固定相、流动相的选择;样品与方法的关系;样品前处理方法	科研思维模式:相似相溶;迂回方式	目标导向策略	(2)(3)(4)
5	色谱新方法的介绍:亲水作用色谱,毛细管电泳,超临界色谱	科研思维模式:交叉领域创新	类比法	(3)(4)
6	色谱的发展趋势:小型,便携,自动	科研兴趣培养:发散思维	想象和发散	(4)

因此,基于学校定位和人才培养特色,针对教学内容与学生思想特点,选择合适的教学方法并设计合理的教学内容,可保证“课程思政”的实施效果。本文从课堂和实验两个环节入手,重视课程思政特色,努力践行“为谁培养人、培养什么人、怎样培养人”的高等教育使命,在传授专业知识的同时探索仪器分析课程的思政建设。

## 1 课堂教学

研究型大学的目标是培养创新型人才,帮助学生获取前沿知识^[8]^。在课堂讲授知识的同时,培养学生的科研兴趣、介绍蕴含专业知识的科研方法是研究型大学本科教学的最重要任务。虽然现代社会知识获取渠道较多,网络上有很多免费资源可供学生学习,但是课堂不可替代的根本原因是教师的科研素养与能力。通过师生交流的方式,使学生的科研能力潜移默化地得到提升。此外,课堂是育人的第一场所,让学生在专业知识的学习中建立正确人生观,培养他们的社会责任和社会认同感,为他们顺利踏入社会提供保障。自然科学的理论体系与社会科学类似,通过类比的讲授方式,让学生领会到自身和社会的关系,最终反映在价值体系中,这种情感的升华是课程思政的精髓所在。在课程内容讲解过程中,穿插该知识点在科研与生活中的应用案例,以国内教师的科研成果作为例子,培养并增强学生民族自信心。例如,在色谱历史讲解中,以20世纪60年代国家在西部化工产业的重大布局为背景,介绍色谱科学、技术与产业在西部地区的发展和应用情况。让学生切实感受到那个年代科学家的奉献精神与科研服务于国家重大战略需求的使命感,相关内容总结于表1。

## 2 实验教学

实验是巩固和实现课堂知识应用的基本途径。纸上得来终觉浅,绝知此事要躬行。在实验课上,如何充分调动学生实验的积极性和创造性,培养学生分析问题和解决问题的能力,是实现知识转化和挖掘思政元素的关键^[9]^。学生通过所学知识进行实际问题的解决,可以获得成就感与专业知识在生活和社会中的具象化体现,并进一步巩固知识并获得举一反三的能力。色谱实验的设计与实施不仅可以让学生“区别”色谱方法,牢记不同方法的特点,还能达到掌握实验技能、解决实际问题和拓展专业知识的目的。此外,本文实验目标还包括使学生获得学科自豪感、使命感和提升自我认可度等。因此,授课教师需精心设计相关色谱实验,贯彻“坚持潜心问道和关注社会相统一,坚持学术自由和学术规范相统一”的精神。

实验讲授的具体内容包括回顾色谱知识、演示溶液配制、安排实验任务和指导学生实验等。例如在色谱知识回顾环节,应用启发、回忆和提问的方式展开,不照搬理论课的内容,根据实验进行知识回顾与启发,侧重讲解课堂没有讲过和无法展示的部分。下面以离子色谱为例说明实验课过程(表2)。课程题目为《某矿泉水中阴离子的含量测定》,该实验在很多高校开设^[10]^。通常按照6个同学为一组,拔尖班2个同学一组组织教学活动,实验总时长4学时。

**表2:** 实验课内容(以离子色谱为例)

序号	模块	具体内容
1	讲解知识点	色谱基本知识,分离的必要性,分离度,柱效评价
	(45 min)	本实验流动相组成与浓度;为什么离子色谱中不需要使用容量瓶精细配制流动相?离子色谱柱为什么采取低交换容量?色谱柱说明书的重要性。
		色谱设备构造:辅助气体的作用、管路和色谱柱材质介绍、抑制器和检测器。本实验设备中抑制器的原理与流路连接技巧,市场上抑制器的类型。
		定性定量方式,具体软件操作
2	示范操作(15 min)	移液枪的使用方法,容量瓶的正确使用方法,标签规范性使用;进样,积分,数据处理
3	学生任务(120 min)	计算取样量,定容,进样(标准曲线6个点和样品3次平行),积分,数据处理,回答问题
4	实验报告撰写	原理、实验内容、数据分析和思考
	(课后45 min)	

在该课程的授课中,教师不仅需在知识点讲解上区别于课堂知识,还应以实验仪器为道具,拓展学生知识点。比如表2中,管路材质是聚醚醚酮(PEEK),让学生在等待色谱流出曲线的时间段查阅并研究此材料的性能;根据仪器连接的抑制器管路,让学生尝试设计并画出抑制器电解用水的其他连接方式并给出理由和性能评价;在讲解过程中不断提出问题,引导学生课后查阅相关文献,进一步拓展和深入研究所学知识(见表3的作业内容)。在实验设计上充分考虑育人环节(表3)。

**表3:** 实验课的知识-能力-思政体系

知识点与相关内容	能力培养	思政点
作业1:查阅一款色谱柱的说明书 作业2:查阅抑制器的发展历程	查阅文献能力	从国内外色谱填料的发展和减少对国外供应链的依赖视角论推动重视“国产化”的必要性
作业3:什么是无接触的电导检测器	科研兴趣培养	习近平总书记指出,核心技术是国之重器,最关键最核心的技术要立足自主创新、自立自强。
作业4:定性方法知多少	总结汇总能力	各有千秋,平分秋色,各有所长;汉语言的魅力,文化自信
操作1:一人配制一个标准溶液,集体获得工作曲线	移液、定容能力	个体对集体的贡献:为了一个共同目标而努力,团结互助是成功的保证。
操作2:两个同学分别配制一个相同标准溶液	检测中的“人员比对”; 误差来源知多少?	个体差异是必然存在的
操作3:戴手套与不戴手套的操作比对	提高微量分析数据准确性的策略; 科研规范性的培养	细节决定成败
数据1:产品定量结果评价	查阅国标对矿泉水的质量规定,对样品进行评判	关心民生,关心健康,关心食品安全;坚持潜心问道和关注社会相统一
数据2:为什么氯离子定量误差大于其他离子	氯离子是人体最丰富的阴离子,人体中阴离子如何测定?启发科研兴趣	基础学科的重要性,学科交叉的重要性。
数据3:本组工作曲线与其他组工作曲线的比较	检测中的“外部比对”	知己知彼,百战不殆
实验报告:实验解决了什么问题,得到什么结论,获得什么启发	思考与总结	习近平:学以致用、用以促学、学用相长。该思想不仅为我们树立了正确的学习观,而且也为我们指明了科学的方法论,对我们端正学风,科学学习,提高学习效果具有重要指导意义。

比如,从实验设计上,以日常生活入手,引导学生讨论色谱仪器的应用领域,将学生关注的目光从课堂转移到社会问题的解决,提升了学生的社会责任感。通过学习和操作实验使用的设备,让学生了解仪器设备的原理与设计思路,体会到工匠精神与“国产化”的重要性,启发学生树立为国家情怀和科技自立自强而工作的信念。在教学方法上,以兴趣和爱好为起点,围绕实际生活中的问题展开。以问题为中心,鼓励学生运用所学知识,在解决问题的过程中不断学习和成长。这样不仅能提升学生的自信心,还能注重培养他们的自我驱动力。在实验操作中,强调科研规范性和创新性相统一,使学生养成认真严谨的实验习惯,提升实验教学的质量。最后,要让每个学生都有事可做,有果可比,有疑可思,有路可进。根据每个同学不同的实验操作任务,启发布置不同的课下思考,形成每个学生不一样的实验报告,避免同一组学生相互重复,相互抄袭。

通过本方法的实施,学生对学科知识的理解得到了进一步巩固和加深,并增强了运用所学知识解决问题的能力。同时,他们对色谱发展、原理、操作及实际生活中的应用、问题的解决和个人能力提升等有了更深的理解和思考。最终,该方法不仅启发学生增强了社会责任感、自我参与感和文化自信,还能使其充分尊重科学、善于运用所学知识。

## 3 结论

本文以课程教学为抓手,强化高校教师育人职能,推动具有中国特色的世界一流大学建设。专业课程实现思政元素挖掘与重构是教学改革的重要途径,教师在授课中不断提升教学水平,才能授人以鱼、授人以渔、授人以欲^[11]^,使学生“学到做人、做事和奉献的道理”,实现知识和价值的融合,思政与教学的相辅相成。在实验教学中,学生留言“不一样的实验体验,最喜欢的实验”,通过手写实验报告,详细记录了自己的感悟与思考。学生对教学模式的认可,实际上也是学生的自我认可和价值体现。所以,在仪器分析课程教学中融入思政元素,不仅丰富了教学内容,还增加了知识的多元性,可启发并增强学生的创新意识和理念,树立人生目标和伟大理想,最终达到立德树人的目的。
